# Addition of lactic acid bacteria to diluted ram semen as vehicle for vaginal inoculation: interaction with seminal microbiota, sperm quality and antibacterial in vitro effect against *Mycoplasma agalactiae*

**DOI:** 10.1186/s12917-026-05536-2

**Published:** 2026-05-06

**Authors:** Raquel Toledo-Perona, Jesús Gomis, Antonio Sánchez, Marion Toquet, Nerea Bailon-Larrañaga, Esther Bataller, Christian De la Fe, Ángel Gómez-Martín

**Affiliations:** 1 Grupo de investigación Agentes Microbiológicos asociados a la reproducción animal (ProVaginBIO), Departamento Producción y Sanidad Animal, Salud Pública Veterinaria y Ciencia y Tecnología de lo s Alimentos, Facultad de Veterinaria, Universidad Cardenal Herrera-CEU, CEU Universities, Valencia, 46115 España; 2 Ruminant Health Research Group, Department of Animal Health, Faculty of Veterinary Sciences, Universidad de Murcia, Murcia, Spain

**Keywords:** Ovine, Caprine, *Lactobacillus*, *Enterococcus*, Probiotic, Semen quality, *Escherichia-Shigella*, Metabarcoding, *16S rRNA*

## Abstract

**Background:**

The use of lactic acid bacteria (LAB) as a probiotic in seminal doses of small ruminants has been suggested due to its antibacterial effects against pathogens such as *Mycoplasma agalactiae* (Ma) and positive effects on ovine vaginal health. The present in vitro study evaluates the antibacterial effect against *Ma* of a wild LAB strain from caprine prepuce and a commercial human probiotic (L3) composed by *Lactobacillus* spp., and evaluates their impact on sperm quality, DNA fragmentation, and the bacterial community composition in the studied conditions. One preputial wild strain (P65) and L3 were tested to assess its antibacterial in vitro potential against *Ma* in semen doses.

**Results:**

In experimental conditions with diluted ram semen, the strain P65, identified as *Enterococcus hirae*, significantly reduced *Ma* concentration from 7.202 to 0.000 log CFU/mL (*p* < 0.05), and L3 from 6.872 to 2.699 log CFU/mL (*p* < 0.001). In addition, experimental condition with P65 suffered a greater *Ma* inhibition and pH reduction over 15 h of incubation (*p* < 0.05). In experimental conditions with LAB, sperm motility exceeded 75% in the presence of *Ma*, with no adverse effects on DNA fragmentation observed. A negative effect on *Ma* was also observed in diluted semen from rams, which could be attributed to the predominance of several species of *Lactobacillus* spp. in the semen diluted without antibiotics. Metabarcoding analyse evidence that *Actinobacillus*, *Lactobacillus*, and *Staphylococcus* were naturally present in the diluted semen studied. Moreover, under conditions with diluted semen and the wild strain P65, no significant increase in opportunistic pathogens as *Escherichia-Shigella* was observed.

**Conclusions:**

This study is the first in vitro report of the antibacterial potential of male reproductive tract LAB against *Ma* and suggests its ecological importance in the reproductive microbiota of small ruminants. The replacement of antibiotics with species-specific LAB in seminal doses of small ruminants should be evaluated in future in vivo studies.

**Supplementary Information:**

The online version contains supplementary material available at 10.1186/s12917-026-05536-2.

## Introduction

*Mycoplasma agalactiae* (Ma) is the main pathogen responsible for contagious agalactia in sheep, with special relevance in dairy small ruminant herds [1–5]. It has been evidenced that mycoplasmosis also has reproductive implications in sheep and goats [6–11] have been identified in buck and ram and their ejaculates [5, 12–14], and their presence has been associated with negative sperm quality impact in diluted semen (DS) from both males species [15, 16] and causing testicular alterations [17]. Therefore, these findings indicate that the real impact of mycoplasmosis in artificial insemination (AI) may be underestimated due to contagious agalactia transmission and potential reproductive repercussions [5].

In this context, broad-spectrum antibiotics such as penicillin, gentamicin, streptomycin, tylosin, spectinomycin, and lincomycin are commonly added to ovine semen diluents to prevent contamination of the seminal dose [18–22]. However, the use of some of these antibacterials has led a significant decrease in the susceptibility of mycoplasma species in small ruminants [5, 23–28]. Moreover, failures in the efficacy of antibiotics combinations in frozen bull semen against *Mycoplasma bovis* have been reported [29]. Regarding ram sperm quality, it is well established that antimicrobials such as gentamicin have a negative impact [22], being necessary to find alternatives to conventional antibiotics use in ovine semen diluents.

It has been suggested that pH reduction could be a potential antibacterial strategy against mycoplasmosis in ruminants. In this regard, the sensitivity of Ma and *M. mycoides* subsp. *capri* to acidic pH in buck DS has been reported [15]. Several lactic acid bacteria (LAB) species exhibit inhibitory effects on the growth of some pathogen’s by acidifying their environment through the production of acetic and lactic acid, along with other antibacterial compounds as bacteriocins, hydrogen peroxide, diacetyl, and carbon dioxide [30–32]. Previousin vitro studies have shown that adding *Lactobacillus* spp. to DS and bovine cervical mucus lowers the pH and negatively affects *M. bovis* viability [33, 34]. In sheep, commercial human strains of *Lactobacillus* spp. have also been shown to reduce vaginal sponge-induced vaginosis without altering the animal’s health status [35] including increased fertility rates [36]. In the aforementioned study, transported and inoculated LAB using AI straws positively modulated the vaginal microbiota, thus offering an interesting alternative to antibiotics. However, it has been suggested that species-specific probiotics are needed to achieve more satisfactory results [37]. In this sense, in vitro studies have reported the antibacterial potential of vaginal and raw sheep and goat milk LAB strains against Ma, suggesting a LAB influence on the success of Ma infections, potentially underestimated [31, 32]. The negative effect observed in vivo on the vaginal abundance of *Mycoplasma* spp. after inoculation of *Lactobacillus* spp. reinforces this hypothesis [36]. Despite of these advances, there is a lack of studies evaluating the potential antibacterial effect of probiotics against Ma infection in ovine semen.

Owing to metabarcoding studies, it is known that LAB with probiotic potential are naturally present in the microbiota of the vagina [35, 36, 38–40], prepuce [38] and semen [21, 39, 40] of healthy small ruminants. Therefore, the presence of LAB in the genital tract could ensure an adequate balance among different bacterial communities. In fact, it has been demonstrated that human ejaculates predominantly containing *Lactobacillus* are associated with good-quality semen [41, 42]. Similarly, a high abundance of this genus in porcine ejaculates positively correlates with sperm quality and reproductive performance in inseminated sows [30]. However, Mocé et al., [39] observed that the processing of buck semen doses caused significant changes in the original ejaculate microbiota, mainly due to semen dilution, potentially reducing the fertilization capacity that semen doses have compared to natural mating. Despite the importance that sperm microbiota may have on sperm quality and fertility, no study has investigated the effect of LAB on sperm quality and their interaction between different bacterial communities in ram DS.

Based on this, the main hypothesis of the present study is that the viability of Ma in ram DS might be negatively affected by adding LAB, potentially replacing antibiotics in seminal doses without negatively impacting sperm quality. Accordingly, the aim of this in vitro study was to evaluate the antibacterial potential of wild LAB isolated from small ruminant’s prepuce and ejaculate against Ma and to compare it with the efficacy of a commercial human probiotic. To achieve this, the viability of Ma and LAB, along with pH fluctuations, were evaluated in ram DS. Additionally, the effects of the pathogen and different LAB strains on sperm quality and sperm DNA fragmentation (SDF) were also determined. Finally, metabarcoding was performed to study the microbiota in ram DS to assess the potential influence of Ma and the LAB strains on the bacterial communities.

## Materials and methods

### Bacterial Strains Isolation and Selection

For the LAB obtention, a total of 90 healthy males were sampled (17 rams and 73 bucks). Samples were obtained from rams and bucks ubicated in insemination centres (IC) and commercial dairy herds (CF) (Table 1). Regarding sanitary conditions, *Coxiella burnetii* circulation was confirmed by q-PCR and serology in the ovine Lacaune herds and in the commercial Murciano-Granadina farm. In that last-mentioned caprine herd, Ma was periodically identified in mastitis cases. No animals showed signs of disease during sample collection. A total of 148 samples were obtained from bucks and rams’ semen (*n* = 53 and *n* = 5, respectively) and preputial swabs (*n* = 73 and *n* = 17, respectively) (Table 2). Before sampling, the external preputial skin was cleaned and disinfected with chlorhexidine 2% following the methodology of Barba et al. [38]. Preputial swabs were obtained by sampling the preputial sac using sterile AMIES PS+VISCOSA swabs (Deltalab, Barcelona, Spain). Semen of animals from IC were collected using artificial vagina with a sterile collection tube [15]. The raw ejaculate samples from males in CF were obtained by electroejaculation [43, 44] in a sterile container. All samples were kept at 4 °C until they were processed.

**Table 1:** Characteristics of the herds analysed and LAB selection

Specie	Breed	Origin	Nº males	NIS	NPS	SS	OD	C
Ovine	Assaf	IC	2	E: *n* = 4 P: *n* = 10	-	-	-	-
Ovine	Manchega	IC	2	P: *n* = 10	-	-	-	-
Ovine	Lacaune	CF	9	P: *n* = 12	P: *n* = 5	-	-	-
Ovine	Latxa	IC	4	E: *n* = 16 P: *n* = 10	E: *n* = 1	-	-	-
Caprine	Boer	IC	1	-	-	-	-	-
Caprine	Murciano-Granadina	IC	3	E: *n* = 1 P: *n* = 5	P: *n* = 1	-	-	-
Caprine	Murciano-Granadina	CF	69	E: *n* = 29 P: *n* = 72	P: *n* = 16	1*	0.62	2.52 × 10^8^

*LAB *lactic acid bacteria, *NIS *number of isolated strains, *NPS *number of potential strains for the experiment, *SS *selected strain for the experiment, *OD *optical density after 20 h incubation, and *C *concentration in CFU/mL after 20 h incubation, *IC *Insemination centre, *CF *commercial farm, *E *ejaculate strain, *P *preputial swab strain. *P65 strain

**Table 2:** Semen and preputial samples, isolated strains and potential LAB strains identified before the in vitro experiment

Type of sample	Nº of samples	NIS	NPS
Caprine_E	53	30	0
Caprine_P	73	77	17
Ovine_E	5	20	1
Ovine_P	17	42	5
Caprine_E + P	126	107	17
Ovine_E + P	22	62	6
Total_E	58	50	1
Total_P	90	119	22
Total_E + P	148	169	23

*LAB *lactic acid bacteria, *E *ejaculate, *P *preputial swab, *NIS *number of isolated strains, *NPS *number of potential strains for the experiment

For the LAB isolation, 100 µL of each ejaculate sample was inoculated on Man, Rogosa, and Shape (MRS) agar (Scharlau, Scharlab S.L., Barcelona, Spain) [45]. In the case of preputial swabs, the swab was inserted into MRS broth for 24 h at 37 °C at 150 RPM. After the preculture, 100 µL were inoculated on MRS agar. The agar plates were incubated for 48 h at 37 °C with 4% CO_2_. The isolates that grew on MRS agar were kept at − 80 °C. After that, these strains were tested on Columbia Agar with 5% sheep blood (BD^™^, Becton, Dicksinson and Company, Madrid, Spain) to rule out haemolytic strains [31, 32]. Potential strains were later inoculated in PH medium to assess their growth capacity in this medium based on previous studies [31, 32]. Only bacterial strains that reached a concentration > 10^8^ CFU/mL were selected for the in vitro experiment.

### Molecular characterization of the wild lactic acid bacteria strain (P65)

Previous to in vitro assay, a bacterial identification for the P65 strain was performed using Sanger technology. Amplification and sequencing of the marker gene * 16S rRNA* was amplifies using the primer pair 27 F/1492R, as it is described in prior investigations [46]. For the above-mentioned analysis, the data was processed using a modified version of the Automated Sanger Analysis Pipeline (ASAP) [47]. Raw chromatogram files were processed using Biopython to obtain FASTQ sequences, which were quality-filtered and merged using the merger tool from European Molecular Biology Open Software (EMBOSS) v.EMBOSS:6.6.0.0 [48]. The resulting merged sequences were used as query for a BLASTn search [49] for species-level identification [32].

In order to obtain a more precise identification of strain P65, the complete genome was analyzed following the previously described methodology. DNA purification checked by sequencing the *16 S rRNA* gene (primers 27 F/1492R) using Sanger technology. Genomic DNA libraries of 600 bp size were prepared, and sequencing was carried out using Illumina NextSeq paired ends (150 × 2 bp) following the protocol described by [32].

### Design of the in vitro experiment

For the study of the interaction of Ma with LAB, 10 experimental conditions (C1-C10) and three control conditions (C11-C13) (Table 3) were prepared with antibiotic-free sperm diluent or specific medium for the isolation of mycoplasma (PH) in Eppendorf-type tubes of 1.5 mL capacity in accordance with previous studies [31, 32], and adapted from a methodology described elsewhere [33, 34]. Wild LAB strain (P65) was tested in three independent replicates of the experimental conditions performed on different days. The number of replicates (*n* = 3) was selected following standard practice in similarly experimental in vitro studies, where three independent replicates are commonly used to ensure reproducibility of treatment effects while maintaining experimental feasibility [15, 31, 33, 34]. As in previous studies [31, 32], conditions using a commercial human *Lactobacillus* spp. probiotic were also included to compare the effects of the P65 strain. The conditions from each replicate were incubated for 15 min (T0), 2.5 h (T2.5) and 15 h (T15). At each experimental time and condition, the viability of Ma and LAB, pH, sperm quality and DNA fragmentation were assessed.

**Table 3:** In vitro conditions composition and least squares means of pH and log CFU/mL of Ma and LAB by time

Condition	Composition	Time	Ma (log CFU/mL)^1^	BAL (log CFU/mL)^2^	pH^3^
1	DS (1,460 µL) + Ma (40 µL)	0	7.204^c^		6.037^g^
1	DS (1,460 µL) + Ma (40 µL)	2.5	6.876^c^		5.740^ij^
1	DS (1,460 µL) + Ma (40 µL)	15	0.000^e^		5.273^kl^
2	DS (1,000 µL) + L3 (500 µL)	0		8.141^efg^	6.050^g^
2	DS (1,000 µL) + L3 (500 µL)	2.5		8.230^defg^	5.663^ij^
2	DS (1,000 µL) + L3 (500 µL)	15		9.202^a^	5.157^l^
3	DS (960 µL) + Ma (40 µL) + L3 (500 µL)	0	6.872^c^	7.664^g^	6.103^g^
3	DS (960 µL) + Ma (40 µL) + L3 (500 µL)	2.5	6.992^c^	8.312^cdefg^	5.767^ij^
3	DS (960 µL) + Ma (40 µL) + L3 (500 µL)	15	2.699^d^	8.816^abcde^	5.237^kl^
4	DS (1,000 µL) + P65 (500 µL)	0		8.388^bcdefg^	6.077^g^
4	DS (1,000 µL) + P65 (500 µL)	2.5		9.064^ab^	5.357^k^
4	DS (1,000 µL) + P65 (500 µL)	15		9.097^ab^	4.807^m^
5	DS (960 µL) + Ma (40 µL) + P65 (500 µL)	0	7.202^c^	7.899^g^	6.063^g^
5	DS (960 µL) + Ma (40 µL) + P65 (500 µL)	2.5	6.911^c^	8.755^abcde^	5.290^kl^
5	DS (960 µL) + Ma (40 µL) + P65 (500 µL)	15	0.000^e^	8.792^abcde^	4.860^m^
6	PH (1,460 µL) + Ma (40 µL)	0	7.098^c^		7.543^ab^
6	PH (1,460 µL) + Ma (40 µL)	2.5	6.968^c^		7.630^a^
6	PH (1,460 µL) + Ma (40 µL)	15	8.497^a^		7.340 ^cd^
7	PH (1,000 µL) + L3 (500 µL)	0		7.961^fg^	7.207 ^de^
7	PH (1,000 µL) + L3 (500 µL)	2.5		8.828^abcde^	6.853^f^
7	PH (1,000 µL) + L3 (500 µL)	15		8.719^abcdef^	6.907^f^
8	PH (960 µL) + Ma (40 µL) + L3 (500 µL)	0	7.172^c^	8.160^defg^	7.100^e^
8	PH (960 µL) + Ma (40 µL) + L3 (500 µL)	2.5	7.026^c^	8.974^abcd^	6.830^f^
8	PH (960 µL) + Ma (40 µL) + L3 (500 µL)	15	8.035^b^	8.855^abcde^	6.840^f^
9	PH (1,000 µL) + P65 (500 µL)	0		7.721^g^	7.170^e^
9	PH (1,000 µL) + P65 (500 µL)	2.5		9.041^abc^	7.143^e^
9	PH (1,000 µL) + P65 (500 µL)	15		8.984^abc^	7.217^de^
10	PH (960 µL) + Ma (40 µL) + P65 (500 µL)	0	7.066^c^	8.106^efg^	7.170^e^
10	PH (960 µL) + Ma (40 µL) + P65 (500 µL)	2.5	7.038^c^	8.814^abcde^	7.197^de^
10	PH (960 µL) + Ma (40 µL) + P65 (500 µL)	15	8.184^ab^	8.139^efg^	7.070^e^
11	DS (1,500 µL)	0			5.967^gh^
11	DS (1,500 µL)	2.5			5.690^ij^
11	DS (1,500 µL)	15			5.253^kl^
12	DS (1,000 µL) + PH (500 µL)	0			6.093^g^
12	DS (1,000 µL) + PH (500 µL)	2.5			5.813^hi^
12	DS (1,000 µL) + PH (500 µL)	15			5.263^kl^
13	PH (1,500 µL)	0			7.557^ab^
13	PH (1,500 µL)	2.5			7.567^ab^
13	PH (1,500 µL)	15			7.443^bc^

*Ma* *Mycoplasma agalactiae* strain PG2, *LAB *lactic acid bacteria, *DS *diluted semen, L3: commercial probiotic inoculum; P65: wild *Enterococcus hirae* strain inoculum; PH: specific medium for *Mycoplasma* spp. growth. T0: 15 min after incubation; T2.5: 2.5 h after incubation; T15: 15 h after incubation. ^1^Standard error of the mean (SEM): 0.55; ^2^SEM: 0.29; ^3^SEM: 0.07. ^a−m^Means in the same columns with different superscripts differs significantly (*p* < 0.05)

#### *Mycoplasma agalactiae* and lactic acid bacteria inoculum

According to the protocol previously described by Toquet and colleagues [31], the reference strain (PG2, NCTC10123) was used to prepare the pathogen inoculum [33, 34]. The strain was cultured in PH broth at 37 °C. A subculture was performed after 48 h of incubation to obtain a Ma inoculum with an approximate concentration in a range of 10^7–8^ CFU/mL [15, 50].

For the LAB inoculum preparation, we followed a previously described protocol to obtain an inoculum (L3) concentration of 3.24 × 10^8^ CFU/mL in PH medium [31, 32] from a lyophilizate commercial probiotic of human origin composed of a mix of three *Lactobacillus* species (*L. crispatus*, *L. acidophilus*, and *L. brevis*) (NS Femibiotic Cinfa). Regarding caprine LAB strain P65 inoculum preparation, a culture of a single colony was done following a previously described methodology [31]. The average concentration of the wild LAB inoculum varied from 7.9 × 10^7^ to 8.4 × 10^8^ CFU/mL.

#### Sperm collection and preparation of diluted semen 

Sperm donors used for the experiment were three adult (two to five years old), healthy rams of mixed-breed meat aptitude, with proven fertility, and trained for regular semen collection using an artificial vagina [15]. The animals belonged to the University research farm (Náquera, Valencia, Spain) from the CEU Cardenal Herrera University. They were housed and fed with a standard balanced diet. The farm population is free of reproductive pathogens such as *Brucella* spp., *Listeria monocytogenes*, *Listeria ivanovii*, Ma, *Chlamydia abortus* and *C. burnetii*. Prior to semen extraction, the preputial external skin was cleaned and disinfected with 2% chlorhexidine according to previous recommendations [51]. For each replicate, ejaculates were obtained from the same three males on different collection days. The three replicates were performed between June and September of 2023. For this, individual artificial vaginas at 40 °C (IMV Technologies® L’Aigle, France) were used in the presence of a female decoy. All the semen collections were carried out by the same investigator. Just after sperm collection, the ejaculate volume, sperm concentration, and pH were calculated. The ejaculate volume was estimated by collecting them in Falcon® (Deltalab, Barcelona, Spain) type graduated semen collection tubes. Sperm concentration was assessed by a cell counter (NucleoCounter® SP-100, ChemoMetec, Allerod, Denmark). The pH value was measured with a calibrated pH-meter (SensION™ + pH3, Hach, LPV2000.98.0002). Ejaculates inclusion thresholds with an optimal volume (0.8–1.2 mL), concentration (2000–3000 million/mL), and pH (5.9–7.3) were used [52]. The raw ejaculate pH mean at T0 was 5.96 and 6.76 for the sperm diluent. Then, ejaculates were prediluted 1:1 with an antibiotic-free sperm diluent (Triladyl® CSS, Minitube) at 30 °C and transported in an airtight isothermal container to the laboratory in less than one hour. Under laboratory conditions, semen from each of the rams was pooled to avoid any individual effect. For the preparation of DS of 1.5 mL, the final sperm concentration in pooled semen was adjusted to 800 × 10^6^ sperm/mL using the same antibiotic-free sperm dilution and distributed into 1.5 mL Eppendorf tubes. Table 3 shows the composition of each experimental condition.

#### Sperm analyses by CASA

The sperm quality of experimental conditions (C1-5, C11, C12) was determined after 15 min (T0), 2.5 h (T2.5) and 15 h (T15) of incubation at 37 °C and 300 RPM. An aliquot (20 µL) of these conditions was taken and diluted (1:20) with antibiotic-free Triladyl® CSS medium. Various sperm parameters (sperm count, kinematics, and morphology) were evaluated using a new generation CASA-Mot system with artificial intelligence to recognize sperm from images (AI Station® v1.2; Sperm Analysis Technologies S.L., Buñol, Spain) in combination with an automatic autofocus microscope (Spermbot®; Sperm Analysis Technologies S.L., Buñol, Spain). As recommended by the manufacturer, 3 µL of semen suspension was loaded into capillary-loaded counting chambers (Spermlide® 20 μm; Sperm Analysis Technologies S.L., Buñol, Spain), and the temperature was maintained at 37 °C by a temperature-controlled stage. The system Spermbot® automatically recorded five fields per sample at 100 frames per s, and the flow rate was maintained at 500 cells/s per field. Videos were recorded for 1 s for each field. The following variables were considered in the results: sperm cell concentration (CON, x 10^6^/mL), total motility (TM, %), progressively motile sperm (PM, %), average path velocity (VAP, µm/s), straight line velocity defined by the straight line between the first and last point on the track (VSL, µm/s), curvilinear velocity measured in a point-to-point reconstitution of the sperm trajectory (VCL, µm/s), straightness index (STR: VSL/VAP × 100, %), linearity (LIN: VSL/VCL × 100, %), wobble (WOB: VAP / VCL × 100, %), normal sperm morphology (NM, %), sperm tail (ST, %), proximal droplet (PD, %) and distal droplet (DD, %) [53–55].

#### Sperm DNA fragmentation (SDF)

The SDF was evaluated using the Sperm-Halomax® commercial kit (Halotech-DNA S.L, Madrid, Spain) based on the sperm chromatin dispersion test, previously reported for this species [56–58]. This staining method enables manual scoring by distinguishing sperm heads based on chromatin response to DNA fragmentation. Sperm with fragmented DNA show a large, faint halo of chromatin dispersion from a residual central core, while unfragmented DNA sperm results in a small or absent halo of chromatin dispersion emerging from a central and compact core. The SDF was evaluated from each DS condition, time and replicate. A total of 50 µL of the sample was analysed after adjusting the concentration to 10 × 10^6^ sperm/mL in sperm dilution medium (Triladyl® CSS). A volume of 50 µL of liquefied agarose was mixed into the tube with 25 µL of the SD. An amount of 2 µL of the agarose-sperm mix was extended on pretreated slides, covered with a 24 × 24 mm coverslip, and placed on a 4 °C metallic plate for 5 min. Subsequently, the coverslip was removed, and each slide was incubated horizontally in 10 mL of lysing solution kit (Sperm-Halomax®) for 5 min. After washing the slide in distilled water for 5 min, nucleoids were dehydrated through a 2-min ethanol series (70 and 100%). Once dried and before the visualization, DNA staining was performed on slides with a 1:1 mixture of fluoGreen® (HT-FG100, Halotech-DNA S.L, Madrid, Spain). This staining method enables manual scoring by discriminating fragmented DNA sperm (large and spotty halos of chromatin dispersion) from those with unfragmented DNA (small and compact halo of chromatin dispersion). Samples were viewed and captured using a EVOS M7000 microscope (ThermoFisher Scientific®). Sperm were counted and classified into fragmented or non-fragmented, and a percentage was calculated based on measuring 300 cells. The analysis of all samples was always carried out by the same person.

#### *Mycoplasma agalactiae* and lactic acid bacteria viability

Bacterial loads (CFU/mL) of Ma and LAB were assessed at T0, T2.5 and T15. Viability of Ma in conditions C1, C3, C5, C6, C8 and C10 (Table 3) was assessed using serial dilutions in PH broth with ampicillin [50] and were plated on PH agar ampicillin-supplemented after an incubation of 72 h at 37 °C in a humidified environment enriched with 4% CO_2_ [15]. Regarding LAB viability, it was determined in conditions C2 to C5 and C7 to C10 (Table 3) by performing serial dilutions in PH broth and plating them on MRS agar [31]. Duplicate plates were used for all dilutions.

#### pH measurement

The pH values were measured with a calibrated pH-meter (SensION™ + pH3, Hach, Loveland, CO, United States) at the three experimental times (T0, T2.5, T15) for all experimental conditions, including raw ejaculate and semen extender. To avoid cross-contamination, the electrode was cleaned and disinfected between experimental conditions [31].

### Microbiota composition analyses

#### Sequencing library preparation and data generation

A total of 32 samples were included for the metabarcoding analysis in order to confirm the *Lactobacillus* spp. presence in L3 and to study their microbial fluctuation in C2 and C3 at T0 and T15. Furthermore, the bacterial community composition was studied from C1, C4 and C5 at T0 and T15 in the three replicates. Finally, a general description of the DS microbiota of rams was performed from C11 between the three replicates at T0. The 16 S ribosomal RNA subunit gene (*16SrRNA*) approach enabled the description and quantification of the microbial diversity and taxonomic phylum-species profiles.

DNA extraction was performed using a commercial kit (MagMAX CORE Nucleid Acid Purification Kit, Applied Biosystems, Thermo Fisher Scientific®, Ref. A32702) [38]. Microbiota profiling was conducted through V3-V4 variable regions of the *16 S rRNA* gene amplification and sequencing. The optimal quality ratios (260/230 and 260/280) together with DNA concentration levels (ng/ µl) were after extraction. The PCR was performed following a methodology previously described [38]. The Illumina Miseq sequencing with 2 × 300 bp reads using v3 chemistry with a loading concentration of 10 pM was used. Amplification was performed after 25 PCR cycles. DNA extraction and amplification blank as negative control, as well as a positive mock community control during library preparation were include as a part of the quality control process.

#### Bioinformatics processing and data analysis

Raw demultiplexed forward and reverse reads were processed using QIIME2 version 2020.111 with default parameters unless stated [59]. DADA2 was used for primer trimming, quality filtering, denoising and pair-end merging, and observed Amplicon Sequence Variants (ASVs) assignation [60]. A Bayesian Classifier allowed the phylotypes taxonomical assignment [61] trained with Silva database version 138 (99% ASVs full-length sequences) [62]. ASVs data was used to calculate alpha diversity metrics: community richness (observed ASVs) and evenness (Pielou’s evenness index). A Generalised Linear Mixed Model was calculated for alpha diversity, particularly the R package NBZIMM v.1.0 [63] (richness) and the R package betareg v.3.1-4 [64] (evenness) to perform diversity comparations. The method MAFFT was used for the ASVs alignment [65] to calculate phylogenetic relations between ASVs Fasttree [66]. ASV and phylogenetic data were used to calculate beta diversity metrics: Unweighted UniFrac, Weighted UniFrac, Jaccard and Bray-Curtis dissimilarities. Permanova and ANOSIM were used to test group significance, and dispersion effects were examined with Permdisp, using a significance level of 0.05 [67].

### Statistical analysis

The statistical differences between species and types of the sample regarding isolation LAB frequency were studied with the EpiInfo software [68] using chi-square cor-rection (Yates) with a 95% confidence level.

Counts of Ma and LAB were transformed as log(1 + C), where C represents the microbial count obtained (CFU/mL) for each analytical condition and organism. Data were analyzed using the General Linear Model procedure (PROC GLM) of SAS® OnDemand for Academics (SAS Institute Inc., Cary, NC, United States), according to the following factorial model: Yijk = µ + Si + Cj + Tk + CTjk + eijk, where Yijk = pH, SDF and log CFU/ml of Ma and log CFU/mL of LAB; µ = mean; Si = sample effect; Cj = effect of analytical conditions; Tk = effect of time; CTjk = effect of the interaction between the analytical condition and time; and eijk = residual effect. Condition (C), time (T), and their interaction (C × T) were treated as fixed effects in the factorial model. Sample (S), representing independent experimental replicates, was included in the model to account for variability among samples. The values obtained were expressed as the mean ± standard error of the mean (SEM). Using the aforementioned statistical model, quality sperm variables were studied: CON (x10^6^/mL), TM (%), PM (%), VAP (µm/s), VSL (µm/s), VCL (µm/s), STR (%), LIN (%), WOB (%), NM (%), ST (%), PD (%), DD (%), SDF (%).

## Results

### Isolation and identification of wild lactic acid bacteria strain (P65)

From the samples obtained, a total of 169 strains were isolated. All of them belong to a collection of the ProVaginBIO investigation group of University CEU-Cardenal Herrera in Valencia, Spain. Only 32 were tested for their growth in PH medium as the other strains haemolysis on blood agar. A total of 23 strains were able to grow in PH medium, but only one preputial caprine strain (P65) was selected, due to the concentration (> 10^8^ CFU/mL) reached 20 h post-incubation, for molecular characterization (Table 1). Regarding the frequency of LAB isolation, the Chi-square test did not show differences between animal species (ovine *vs.* caprine). In contrast, there were significant differences (*p* < 0.05) concerning the type of sample (preputial swab *vs. *raw ejaculate), where the frequency of LAB detection in the preputial swabs was significantly higher than in semen samples.

Based on the BLASTn results, strain P65 with a sequence length (pb) of 1,354 was identified as *Enterococcus hirae*. This strain was deposited under the Budapest Treaty in the Spanish Type Culture Collection as an International Depositary Authority (based in Building 3 CUE, Parc Científic Universitat de Valencia, C/ Catedrático Agustín Escardino, 9, 46980 Paterna (Valencia) SPAIN). The deposit number assigned was CECT 31,332.

### Effects of the variables on *Mycoplasma agalactiae*, lactic acid bacteria viability and pH

The control conditions C11-C13 were tested on sheep blood agar plates, MRS agar plates and PH agar plates at all time points and always came out negative. Table 3 details the evolution of the pH and the viability of Ma and LAB from each in vitro condition for the three measured time points over the three replicates. The in vitro results regarding the effects on Ma viability (log CFU/mL) showed that the condition, time and the interaction between condition and time had a significant effect (*p* < 0.05). Regarding LAB viability (L3 and P65), the factor time showed significant differences (*p* < 0.05), where LAB log CFU/mL at T2.5 and T15 was significantly higher than at T0. The condition does not affect the LAB viability independently of the used medium (DS or PH) or the presence of Ma. Finally, all the factors studied (replica, condition, time, and the interaction between condition and time) were statistically significant (*p* < 0.05) for pH values. The average pH for all three times in C11, C12 and C13 was 5.64, 5.72 and 7.52, respectively.

#### *Mycoplasma agalactiae* viability and pH

Regarding the conditions with DS, the concentration of Ma significantly decreased in all three conditions at T15: it was null for C1 (DS + Ma) and C5 (DS + Ma + P65) and 2.699 for C3 (DS + Ma + L3) (*p* < 0.05). Significant differences were observed for Ma viability at T15 in C3 with C1 and C5 (*p* < 0.05). However, no differences were identified between C1 and C5 at any time. Regarding pH in those same conditions, a significant decrease over time was also observed (C1, C3, and C5; Table 3) (*p* < 0.05). Moreover, conditions with P65 (C4 and C5) had a significant lower pH than C1, C2 and C3 at T2.5 and T15 (C1-C5; Table 3) (*p* < 0.05). The pH in all conditions with DS was significantly lower than conditions with PH at any time point (*p* < 0.05).

On the contrary, in conditions with PH medium (C6, C8 and C10) Ma increased significantly its viability at T15 (*p* < 0.05). However, a significant lower growth of Ma was only observed at T15 in condition C8 (with L3) with respect to C6 (Table 3) (*p* < 0.05). Despite this, there were no significant differences in the pathogen’s viability depending on the type of LAB at any time point in PH medium. A pH reduction was observed over time in most conditions with PH medium (*p* < 0.05) except the conditions with P65 (C9 and C10). Nevertheless, at all times, the pH was significantly higher in C6 (PH + Ma) compared to the conditions with LAB and Ma (C8 and C10). Additionally, the pH of C8 was significantly lower than that of C10 at T2.5 and T15 (Table 3).

With respect to the pH values of the control conditions, PH medium (C13) showed a higher pH than C12 (PH + DS) and C11 (DS) (*p* < 0.05) at all times. Additionally, no significant differences were observed between conditions with only Ma (C1 and C6) and the control conditions (C11 and C13) (Table 3).

#### Lactic acid bacteria viability and pH

Overall, increase in LAB viability was observed throughout time (T0 *vs.* T15, *p* < 0.05), with no differences between T2.5 and T15. In condition C4 (DS + P65), the pH decreased significantly over time (*p* < 0.05) in contrast to C9 (PH + P65), which did not show a reduction. For the rest of the conditions with LAB, there was a significant reduction in pH over time (Table 3) (*p* < 0.05). The strain P65 produced a significantly greater pH reduction in DS (C4) compared to L3 (C2) at T2.5 and T15 (*p* < 0.05). Nevertheless, the condition with L3 in PH medium (C7) showed a lower pH at T2.5 and T15 compared to the condition with the wild strain P65 (C9) (*p* < 0.05). The conditions with DS and LAB (C2 and C4) showed a significantly greater reduction in pH at T2.5 and T15 compared to the conditions in PH medium (C7 and C9) (*p* < 0.05).

### Metabarcoding results: bacterial composition per condition and time

Samples were subsampled up to 18,510 reads to even sample size and make quantitative comparisons. Reads were truncated at the position when the 25th percentile Phred score felt below Q20: 300 bp for forward reads and 252 bp for reverse reads. A total of 1,891,040 pair-end reads were obtained. After quality filtering, trimming and denoising steps, 1,533,088 reads remained. The average sample size was 52,529 reads (min: 6,348 reads, max: 102,956 reads). Paired-end reads were merged and after chimera removal, 1,492,795 merged reads were used for phylotype calling DADA2 [60]. Singletones and doubletones were removed before diversity analysis. Finally, 260 phylotypes were detected. For the DS description, only two of the three replicates were used, as the third lacked sufficient reads for inclusion in the results.

#### Diversity analysis: alpha and beta diversity

**Fig. 1:**
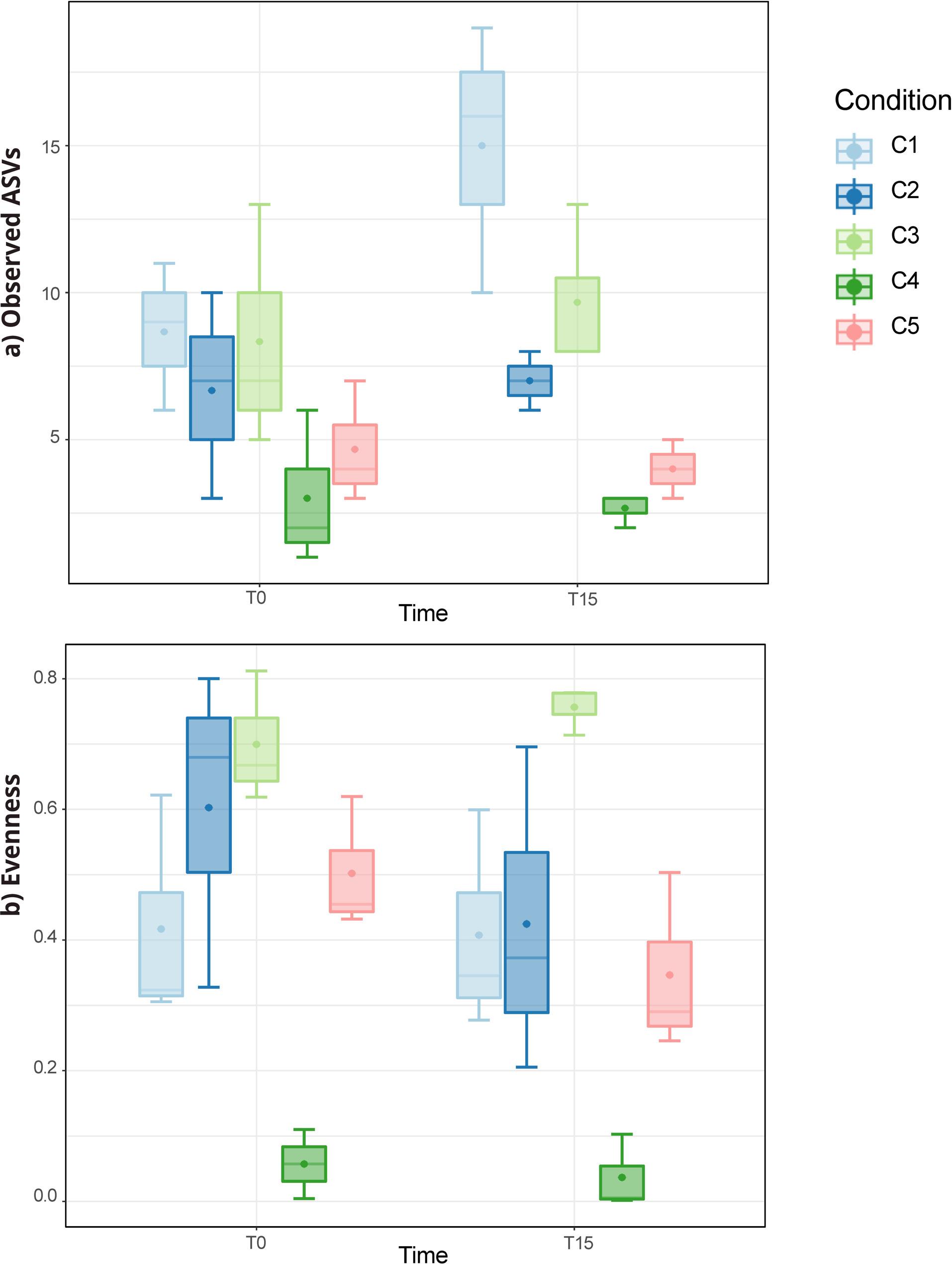
Alpha diversity analysis of observed ASVs (richness) and Pielou's evenness index in the comparison between conditions over time. C1: condition 1 with diluted semen and Mycoplasma agalactiae PG2; C2: condition 2 with diluted semen and L3; C3: condition 3 with diluted semen, Mycoplasma agalactiae PG2 and L3; C4: condition 4 with diluted semen, Mycoplasma agalactiae PG2 and P65; C5: condition 5 with diluted semen, Mycoplasma agalactiae PG2 and P65; T0: 15 min after incubation; T15: 15 h after incubation

The results from the alpha and beta diversity analyses are described in Supplementary Table S1-S3. Regarding alpha diversity, represented in Fig. 1 for richness and evenness, significant differences (*p* < 0.05) were observed between conditions and experimental times. The condition showed a significant effect in the community richness (*p* < 0.01). Condition 1 (C1), which had the highest richness values at T0 and T15 (9 and 16, respectively observed ASVs), was the only experimental condition that increased the community richness over time (*p* < 0.01). Conditions C4 (T0 = 3 and T15 = 2.67 observed ASVs) and C5 (T0 = 4.67 and T15 = 4 observed ASVs) showed the lowest richness index at both time points, decreasing over time. At T15, the observed ASVs for C4 were significantly lower than C3 (*p* < 0.05), and both P65 conditions (C4 and C5) presented significantly fewer ASVs than C1 (*p* < 0.05). Pielou’s evenness index showed C3 as the condition with the highest evenness at T0 (0.67) and T15 (0.78), being significantly higher than other conditions at T15 (*p* < 0.05). Similar to richness results, the conditions C4 and C5, specifically C4, presented the lowest values of evenness in both times (T0 = 0.06 and T15 = 0.01 evenness index). Moreover, the comparation between condition and time showed that C4 had lower evenness values compared to the other conditions in T0 and a T15 (*p* < 0.01). Finally, evenness index of C2 decreased significantly between times (*p* < 0.05).

**Fig. 2:**
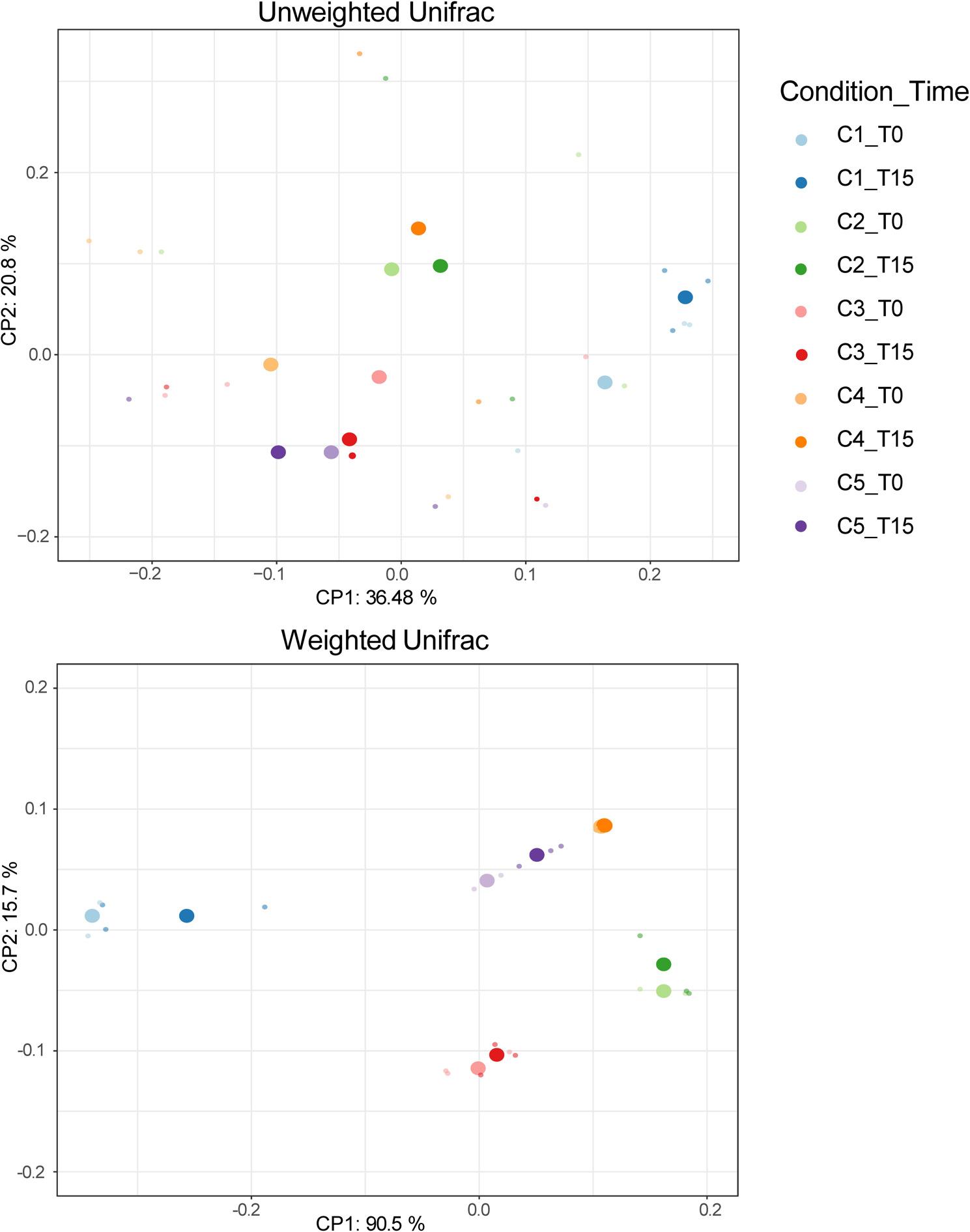
Beta diversity analysis (PCoA plots Weighted and Unweighted Unifrac) in the comparison between conditions over time. C1: condition 1 with diluted semen and *Mycoplasma agalactiae* PG2; C2: condition 2 with diluted semen and L3; C3: condition 3 with diluted semen, *Mycoplasma agalactiae* PG2 and L3; C4: condition 4 with diluted semen, *Mycoplasma agalactiae* PG2 and P65; C5: condition 5 with diluted semen, *Mycoplasma agalactiae* PG2 and P65; T0: 15 min after incubation; T15: 15 h after incubation

For beta diversity, Weighted UniFrac distances according to PERMANOVA test revealed that the effect of the factor time significantly affects the microbial community structure (*p* < 0.05) (Fig. 2).

#### General Taxonomic Composition

**Fig. 3:**
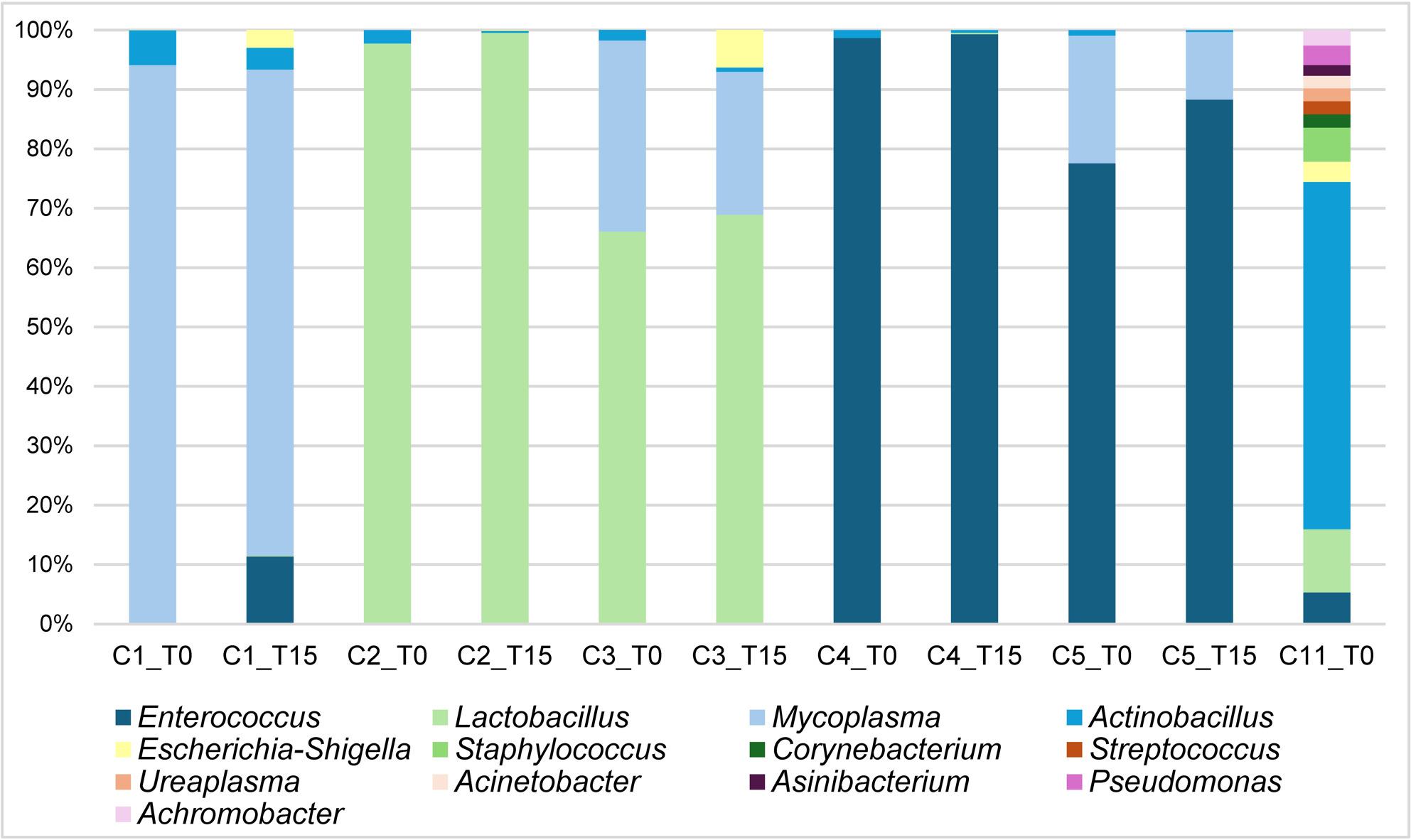
Relative mean abundance of genus-taxa from each condition including both experimental times. C1: condition 1 with diluted semen and *Mycoplasma agalactiae* PG2; C2: condition 2 with diluted semen and L3; C3: condition 3 with diluted semen, *Mycoplasma agalactiae* PG2 and L3; C4: condition 4 with diluted semen, *Mycoplasma agalactiae* PG2 and P65; C5: condition 5 with diluted semen, *Mycoplasma agalactiae* PG2 and P65; C11: condition 11 with diluted semen; T0: 15 min after incubation; T15: 15 h after incubation

**Fig. 4:**
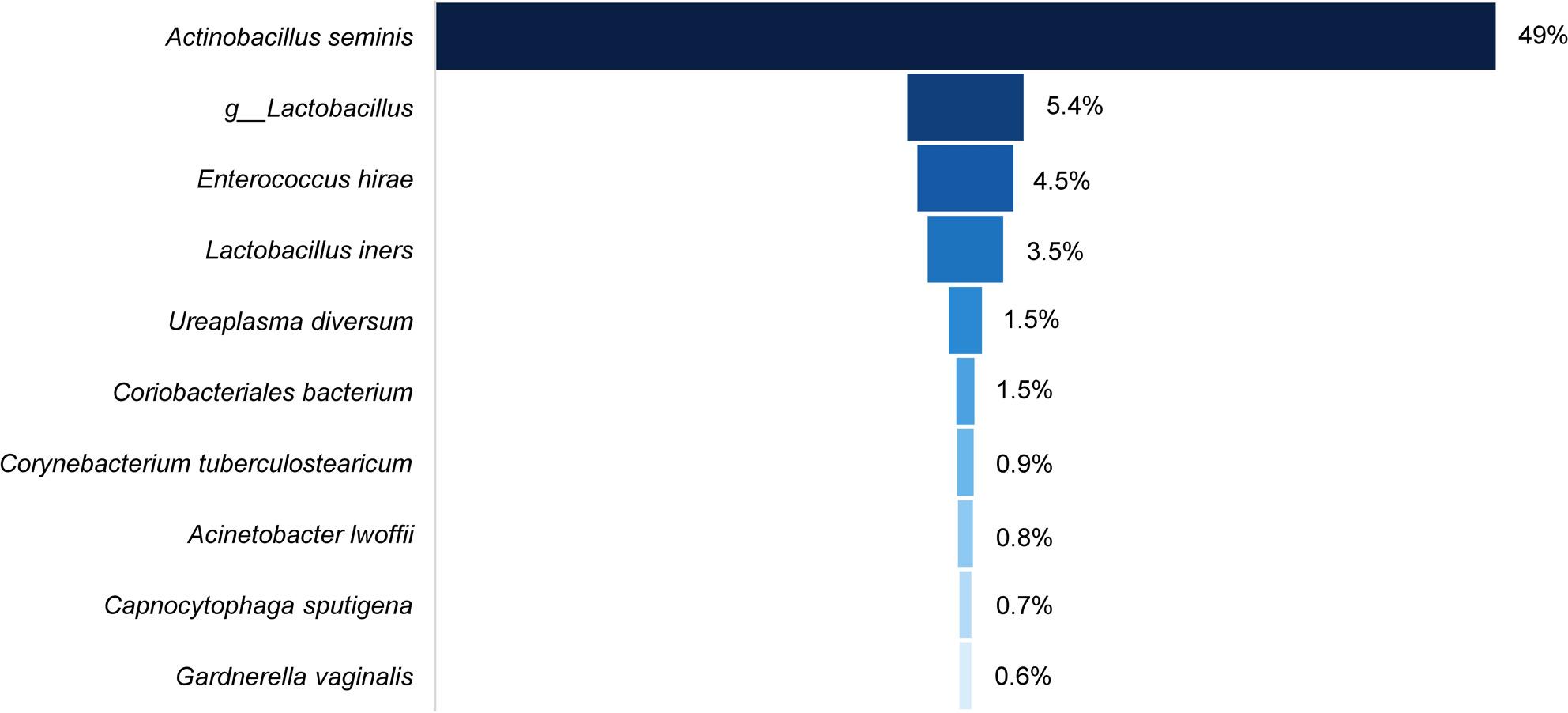
Relative abundance of bacterial species detected in condition 11 (C11, diluted semen) from both replicates. *g*: bacterial genera.

In relation to the ram’s DS microbiota description (C11), *Actinobacillus*, *Lactobacillus*, *Staphylococcus* and *Enterococcus* were the genera with highest relative abundance (RA) (Fig. 3). The species with RA > 1% are shown in Fig. 4.

**Fig. 5:**
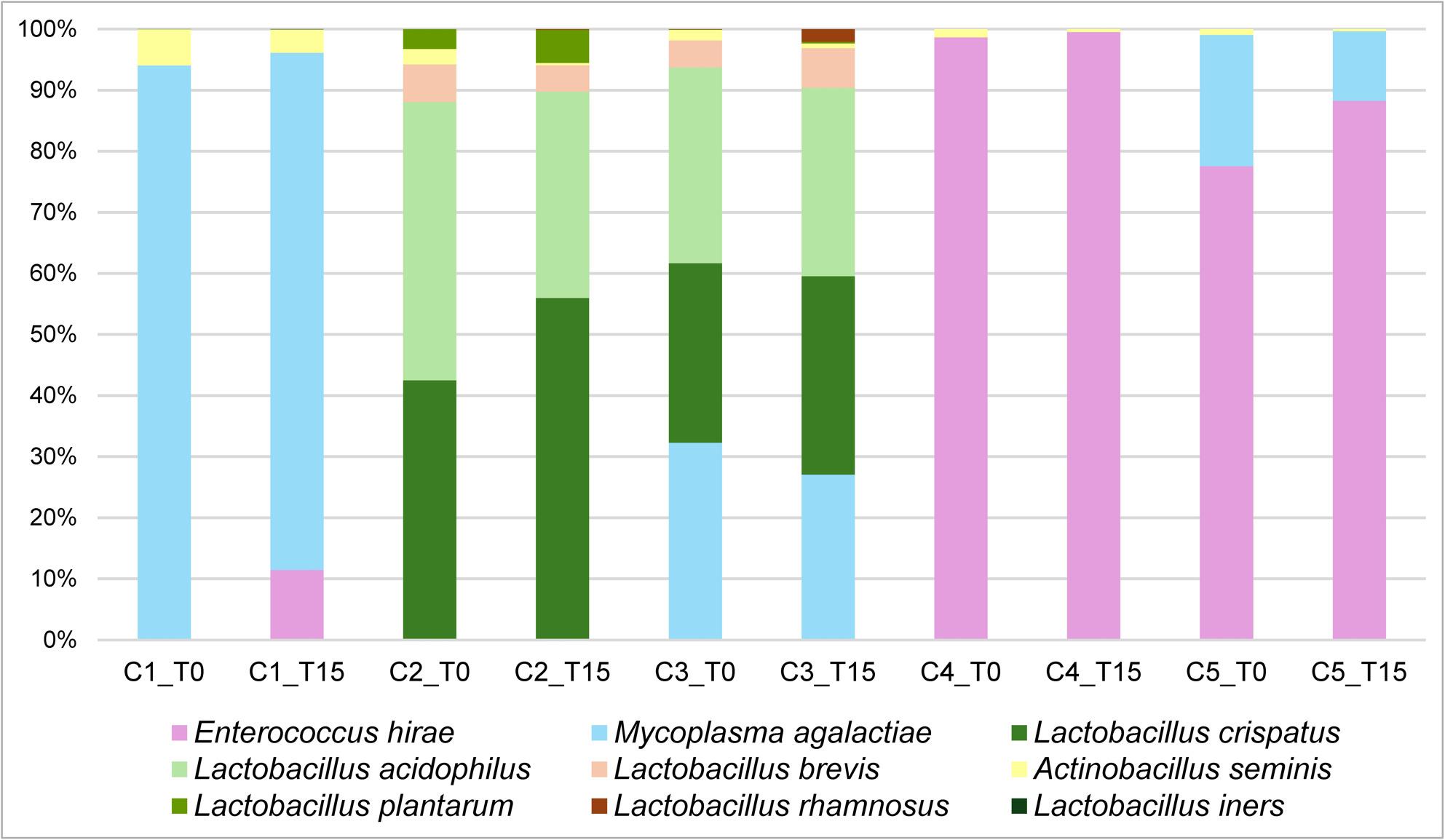
Relative mean abundance of specie-taxa from each condition including both experimental times. C1: condition 1 with seminal dose and *Mycoplasma agalactiae* PG2; C2: condition 2 with seminal dose and L3; C3: condition 3 with seminal dose, *Mycoplasma agalactiae* PG2 and L3; C4: condition 4 with seminal dose, *Mycoplasma agalactiae* PG2 and P65; C5: condition 5 with seminal dose, *Mycoplasma agalactiae* PG2 and P65; T0: 15 min after incubation; T15: 15 h after incubation

Regarding the experimental conditions composed by DS, the mean abundance of taxa at the genus level, depending on the experimental condition over time, is described in Fig. 3, and for species in Fig. 5. The genus *Enterococcus*, and particularly the species *E. hirae*, was the most abundant for the conditions C4 (T0 and T15 = 99%) and C5 (T0 = 78%; T15 = 88%) (Fig. 5). This genus showed a significantly increase at T15 comparing to T0 in conditions C1 and C3 (*p* > 0.05). *Lactobacillus* showed the highest RA in conditions C2 (T0 = 98%; T15 = 99%) and C3 (T0 = 66%; T15 = 69%). In this regard, metabarcoding analysis revealed the presence of the three main LAB species in conditions containing L3 (C2 and C3) at both T0 and T15. The species *L. acidophilus* showed the highest RA at T0 (C2_T0 = 41%; C2_T15 = 28%; C3_T0 = 32%; C3_T15 = 28%), followed by *L. crispatus* (C2_T0 = 38%; C2_T15 = 46%; C3_T0 and C3_15 = 29%), which its RA increased at T15 in C2. The RA for *L. brevis* decreased for C2 (T0 = 6%; T15 = 4%) and increase for C3 (T0 = 4%; T15 = 6%) over time. Other *Lactobacillus* species identified with a lower RA (< 5%) were *L. plantarum*,* L. rhamnosus* in C2 and C3, and *L. iners* in C1 (RA < 0.1) with a significant increase at T15 (*p* < 0.01) (Fig. 5). In continuation with the most relevant observed taxon, *Mycoplasma*, specifically Ma, was the most abundant in C1 at both times (T0 = 94%; T15 = 82%) (Fig. 3). In the conditions with *Mycoplasma*, a reduction of the genus was observed in C3 (T0 = 32%; T15 = 28%) and at C5 (T0 = 21%; T15 = 11%) (Fig. 3). As seen in Figs. 3 and 5, in all the conditions, the pathogen’s RA decreased at T15. Interestingly, *Actinobacillus seminis* was present in the DS of all the conditions at both times (RA < 6%), where a significant decrease in its RA was observed over time (*p* < 0.05). Finally, *Escherichia-Shigella* RA increased significantly in C1 and in C3 (*p* < 0.05).

### Sperm quality

After interpreting the results, it was decided to exclude T15 from the statistical analysis of the sperm quality study since spermatozoa from all experimental conditions were static after such a long conservation at 37 °C. The interaction condition per time did not show any significant effect on the studied variables. The variables CON, ST and PD were not significantly affected by the factors studied. Time significantly influenced two sperm kinetic parameters (TM and VSL), and one sperm morphology parameter (DD %) (*p* < 0.05) (Table 4).

**Table 4:** Least squares means of sperm quality parameters over time

Sperm quality variables	Time (h)			SEM	
	T0 h		T2.5 h		
CON (x10^6^/mL)	833.90		851.45		36.58
TM (%)	87.23^a^		81.06^b^		1.46
PM (%)	91.11		89.26		0.82
VAP (µm/s)	164.41		157.58		3.56
VSL (µm/s)	98.89^a^		86.26^b^		4.33
VCL (µm/s)	342.90		330.37		10.28
STR (%)	53.04		50.14		1.43
LIN (%)	26.62		25.40		0.92
WOB (%)	45.92		46.37		1.27
NM (%)	92.91		93.57		0.39
ST (%)	0.00		0.01		0.01
PD (%)	1.20		1.30		0.10
DD (%)	5.22^a^		3.99^b^		0.36

*SEM *Standard error of the mean. ^a−c^Means with different superscripts in a row differ significantly (*p* < 0.05). T0: 15 min after incubation; T2.5: 2.5 h after incubation. *CON *concentration, *TM *total motility sperm, *PM *progressive motility, *VAP *average path velocity, *VSL *straight line velocity, *VCL *curvilinear velocity, *STR *straightness index, *LIN *linearity index, *WOB *wobble, *NM *normal sperm morphology, *ST *sperm tail, *PD *proximal droplet, *DD *distal droplet

The condition factor only had a significant effect on TM % (*p* < 0.05) (Table 5). Specifically, C3 (DS + Ma + L3) and C5 (DS + Ma + P65) showed the lowest values achieved for TM %, showing no significant differences between them. Considering the effect of each experimental time, TM % in C3 and C5 was significantly lower compared to the control (C11). Despite of, at T2.5 the condition with P65 showed a higher TM % (79.21%) than the condition with L3 (75.683%).

**Table 5:** Least squares means of total motile sperm according to the conditions studied

Condition	Condition description	TM (%)
C1	DS + Ma	90.28^a^
C2	DS + L3	83.81^ab^
C3	DS + Ma + L3	75.68^c^
C4	DS + P65	84.31^a^
C5	DS + Ma + P65	79.21^bc^
C11	DS	89.91^a^
C12	DS + PH	85.83^a^

^a−c^Means with different superscripts differ significantly (*p* < 0.05). Standard error of the mean (SEM): 2.728. *TM *total motility sperm, *DS *diluted semen, *Ma* *Mycoplasma agalactiae* strain PG2; L3: commercial probiotic inoculum; P65: wild *Enterococcus hirae* strain inoculum; PH: specific medium for *Mycoplasma* spp. growth

### Halomax

**Fig. 6:**
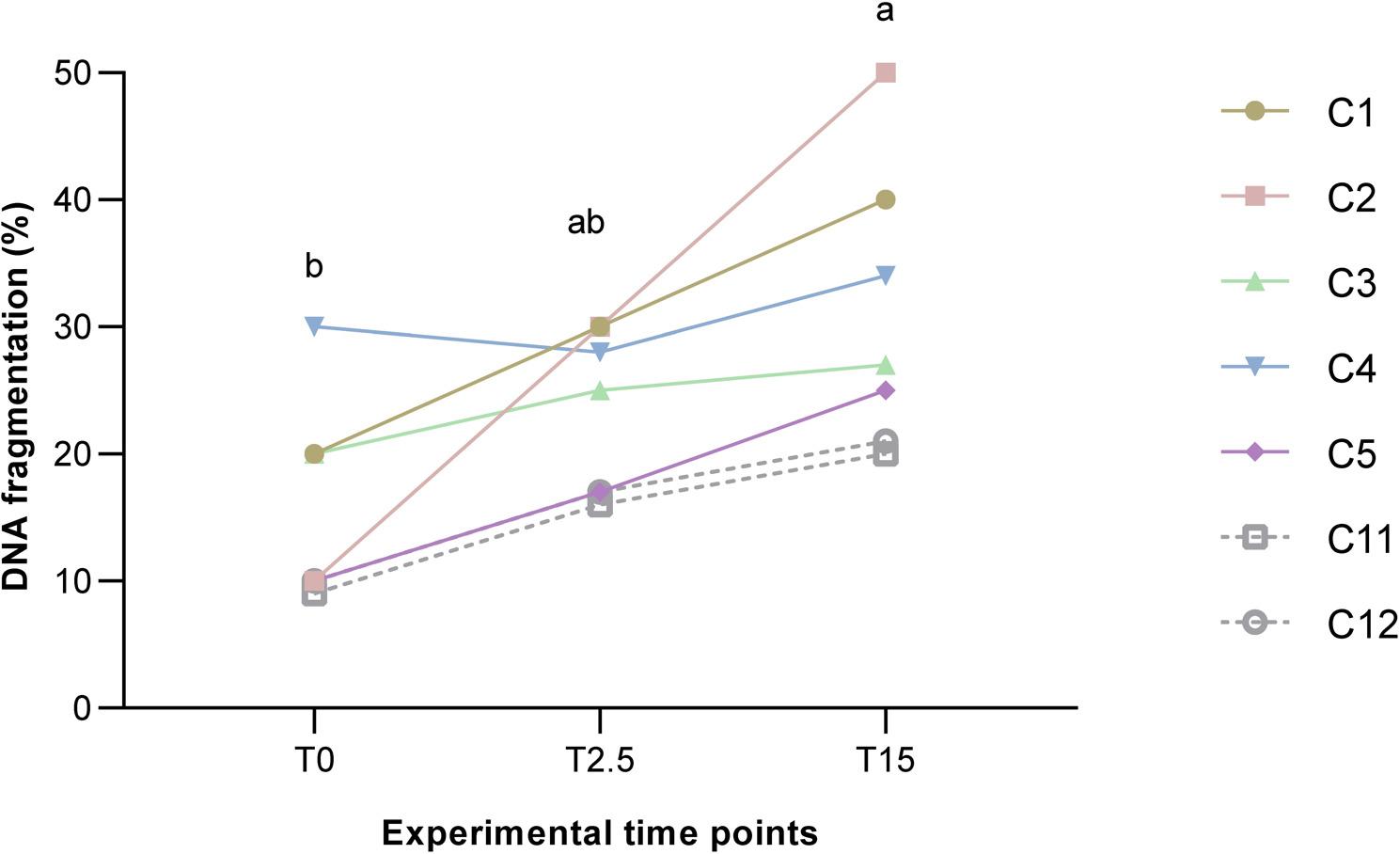
Sperm DNA fragmentation results at different experimental conditions and times. Least squares mean (LSM) at T0 (15 min after incubation): 6.48; T2.5 (2.5 h after incubation): 8.89; T15 (15 h after incubation): 16.02. ^a−b^LSM with different superscripts differ significantly (*p* < 0.05). Standard error of the mean (SEM): 1.532. C1: condition 1 with diluted semen and *Mycoplasma agalactiae* PG2; C2: condition 2 with diluted semen and L3; C3: condition 3 with diluted semen, *Mycoplasma agalactiae* PG2 and L3; C4: condition 4 with diluted semen, *Mycoplasma agalactiae* PG2 and P65; C5: condition 5 with diluted semen, *Mycoplasma agalactiae* PG2 and P65; C11: condition 11 with diluted semen; C12: condition 12 with diluted semen and PH medium. T0: 15 min after incubation; T2.5: 2.5 h after incubation. T0: 15 min after incubation; T2.5: 2.5 h after incubation

Sperm samples processed for DNA fragmentation visualization showed two different spermatozoa population: fragmented sperm (sperm nuclei that exhibited medium or large peripheral halos of diffusion of chromatin spots) and unfragmented (sperm nuclei remained compact or displayed small halos of chromatin spreading). Only the variable time was significant, showing a significant increase (*p* < 0.05) of the % SFD between T0 (6%) and T15 (16%) but not compared to T2.5 (9%) (Fig. 6).

## Discussion

The present in vitro study is the first to describe the microbiota of antibiotic-free DS in rams and examine the effects of LAB addition, as well as the influence of Ma on it. It also reports the effect of commercial LAB strains and a wild LAB strain (P65), isolated from a buck’s reproductive tract, against Ma preserving optimal sperm quality parameters. Therefore, these results elucidate an alternative strategy antibiotics addition to seminal doses and a novel approach to indirect intravaginal probiotic inoculation in small ruminants and other animal species by using DS enriched with LAB.

It is well established that antibiotics are commonly added to ovine semen extenders to reduce microbial contamination in seminal doses [19–22]. However, the extensive use of some of these antibacterials has caused a significant decrease in the susceptibility of Ma [23, 24, 27, 28]. This highly contagious pathogen with reproductive tropism [5], has been detected in small ruminant ejaculates [12–14]. Moreover, the negative impact of antibacterials on sperm quality in ovine has been described [22]. As an alternative, the use of probiotics, such as LAB, in AI semen doses has been suggested in ovine [21], however it had not yet been evaluated. The natural presence of LAB in the reproductive tract of male and female small ruminants [21, 32, 38, 40] and the in vitro [31, 32] and in vivo [36] antibacterial potential of wild vaginal LAB strains against *Mycoplasma* spp. have been reported. Nevertheless, there are no studies that have looked for this type of wild strains in the male urogenital tract. Moreover, various studies have demonstrated the beneficial use of LAB as an alternative to antibiotics in DS in different livestock species such as cattle and pigs [33, 69]. In this sense, it has also been reported that the use of commercial human probiotics based on *Lactobacillus* reduce thein vitroviability of *M. bovis* in both cervical mucus and diluted bovine semen [33, 34]. Nevertheless, there are currently no reports evaluating the antibacterial potential of any strain of LAB of small ruminants against Ma infection in semen. Our results confirm the presence of LAB in the preputial and ejaculates of small ruminants. Despite the low abundance of LAB in the vaginal microbiota of domestic ruminants [21, 70–72], *Lactobacillus* has been described as one of most prevalent bacteria genera in bucks’ semen by metabarcoding [40]. Moreover, species from *Lactobacillus* have also been identified in preputial swabs from healthy adult rams [21, 38]. The significantly higher frequency of LAB isolation preputial swabs compared to raw semen from bucks and ram might be related to the environmental microbiome and its influence on the microbiota of other anatomical sites as the prepuce [73]. Therefore, LAB are an essential bacterial community, which naturally resides in the seminal and preputial microbiome of small ruminants. However, during the dilution of the ejaculate with extenders to obtain commercial semen doses, it is possible that the abundance of these LAB might be reduced. In fact, it has been pointed out that the processing of semen doses in bucks for AI negatively impacts sperm quality and modifies the diversity and composition of the raw ejaculate microbiota [39]. Despite this, LAB were naturally present in the microbiota of the DS (C11) used in our study (Fig. 4). Nonetheless, little is known about the biological role of LAB in the reproductive tract of bucks and rams.

In this study, the commercial probiotic (L3) and the wild strain isolated from a buck’s prepuce (P65) selected for the in vitro challenge against Ma showed an antibacterial effect. Specifically, it was evidenced that conditions with Ma in DS medium (C3, and C5) showed a significant reduction in Ma log CFU/mL at T15 compared to T0 and T2.5. In the same way, other in vitro studies [33, 34] also observed antibacterial potential of a commercial human probiotic (L2), similar to L3, against *M. bovis*. This greater bactericidal effect observed in P65 could be due to its specificity for the genital tract in males of small ruminants. In fact, the well-known host-specific nature of LAB [74] could enhance adhesion and action potential of indigenous LAB strains when used in their appropriate animal species [31, 32]. Additionally, in presence of P65, the proliferation of enterobacteria, such as *Escherichia-Shigella* was not observed by metabarcoding (Fig. 4). It suggests a possible antibacterial effect against other bacterial groups, such as *Enterobacteriaceae*, commonly causing seminal doses contaminations in ovine [20]. Besides, this bacterial group has been associated with vaginosis following the use of intravaginal devices for estrus synchronization in sheep [35]. In fact, Toquet et al., [36] has previously reported a reduction of *Enterobacteriaceae* following LAB intravaginally inoculation. Together with the present study’s findings, LAB may represent an alternative for controlling enterobacteria implicated in reproductive diseases in small ruminants, potentially exerting a negative impact on fertility.

Wild LAB strain P65 also showed the greatest pH reductions in DS. Indeed, the pH values in the presence of P65 were significantly lower than with L3 regardless of the presence or absence of Ma, as well as compared to the favourable condition (C1) and control (C11). The great acidification capacity of P65 may allow the inhibition of other pathogenic of *Mycoplasma* spp. Although these bacteria can produce bacteriocins, hydrogen peroxide, diacetyl, and carbon dioxide to control pathogen growth [71, 72, 75, 76], the acidifying capacity of LABs producing lactic acid has been described as an effective strategy for inhibiting some pathogens [31, 33, 34]. One possible reason why strain P65 can lower the pH more than L3 in ram DS with a greater bactericidal effect could be its natural adaptation to the genital environment of male small ruminants. More specifically, in the human male reproductive system, *E. hirae* can acidify the environment producing acid from melibiose and sucrose [77]. In our conditions, this fact could contribute to the reduction of pH in the DS conditions, creating a hostile environment for Ma. Interestingly, it should also be considered that P65 (*E. hirae*) was isolated from an asymptomatic herd with a history of contagious agalactia due to Ma. Therefore, it might be the result of natural selection in the bacterial ecology of the herd. The presence of *E. hirae* strains, with bacteriostatic and bactericidal effects against Ma, after outbreaks of contagious agalactia has also been reported in ewe’s milk [31]. The authors also suggested that the presence of certain LAB strains in a host could negatively influence the viability of Ma in vivo. The antibacterial capacity of *E. hirae*, isolated from dairy domestic ruminant’s products, has also been reported against other bacteria, such as *L. monocytogenes*, through in vitrobacteriocin production [78, 79]. However, in addition to the alternative mechanisms of action mentioned above, the possible presence of bioactive peptides should be considered as a potential antimicrobial pathway of LAB [80]. Further mechanistic validation is necessary to elucidate the possible mechanisms underlying this bactericidal effect. The antibacterial potential of LAB against Ma shown in this study, without negatively affecting sperm quality, highlights the addition of LAB to seminal doses as an alternative to antibiotics for a possible control of reproductive infections caused by Ma or other reproductive pathogens if further studies are pursued.

Concerning the LAB viability, an increase in log CFU/mL was observed over time in conditions with L3 and P65, which is in accordance with other in vitro studies in small ruminant milk [31], cervical mucus and diluted bovine semen [33, 34]. This finding would reinforce the idea that ovine DS is a suitable environment that could favour LAB. Considering this, and the antibacterial potential of LAB against Ma, semen doses could potentially serve as a vehicle for the transmission of LAB to the ovine vagina, without any adverse effect on sperm quality. In this sense, recently, a double intravaginal inoculation of sheep with preserved LAB, administered before and after inserting intravaginal sponges via AI straws, has been shown to improve fertility and vaginal microbiota health by reducing *Mycoplasma* spp. abundance [36]. Our results suggest the potential for future studies to evaluate the co-inoculation of LAB with DS, which may contribute to a simplified inoculation protocol. Additionally, the present results also showed an intrinsic inhibitory effect of ram DS against Ma even without antibiotics (C1), previously described in buck DS with Ma [15] or in bull DS with *M. bovis* [33]. This could be associated with the acidic pH of the DS. It is known that mycoplasmas are particularly sensitive to pH variations in the environment [81, 82]. In our study, the reduction in Ma viability in C1 could be attributed to the sperm cells in the diluent decreasing the pH of the medium. This is consistent with findings from previous in vitro studies on goat DS infected Ma and *Mycoplasma mycoides* subsp. *capri* [15], where the pH of raw ejaculate (pH ≥ 7) was higher than that of DS (pH ≤ 6). In our study, DS in ram also presented a lower pH values in comparison with raw ejaculate. The acidification of the DS medium could be due to the metabolic activity of spermatozoa [83] or derived from the glycolytic activity of sperm in the extender, which reduces the viability of *Mycoplasma* spp. in domestic ruminant DS [15, 33]. However, an antibacterial effect of the microbiota naturally present in DS should be considered. In this sense, metabarcoding results evidenced a natural abundance of LAB in the ram DS (Fig. 5), which could be impacted by the routinely use of antibiotics in DS. We suggest that synergistic effects may also occur with other LAB present in DS (C11), or through the production of metabolites and antibacterial peptides specific to their metabolism within DS. Nevertheless, this antibacterial effect was observed in DS, was not seen with any of the LAB strains in conditions with PH medium. This could be because the mycoplasma-specific PH medium is merely affected by pH acidification, altering the proper functioning of antimicrobial mechanisms exhibited by LAB and allowing the pathogen to have a greater ability to survive [31]​.

Although the beneficial effects of LAB in semen have been reported, there are no studies to date that have described their impact on the sperm quality of small ruminants. In pigs and humans, it has been seen that the presence of *Lactobacillus* spp. offers beneficial effects on semen quality [30, 41, 42, 84]. In addition to that, LAB have also been used as dietary supplements to improve sperm parameters in dogs [85] and humans [86, 87]. In the case of boar seminal doses, the use of LAB as antibacterial is seminal doses has been previously evaluated. In that study, authors observed that the addition of LAB in boar seminal doses, without antibiotics and stored at 17 °C for 168 h, harmed sperm quality compromising their suitability as an alternative to the use of antibiotics in boar seminal doses [88]. In ram DS, our results indicate that the addition of LAB (P65 and L3) in the absence of Ma had no negative effect on any sperm quality parameters. However, the conditions where LAB (P65 and L3) and Ma were combined (C3 and C5) did show a significant reduction in TM % at T2.5. Despite this, the TM % at T2.5 in both conditions was higher than 75% and 79% respectively, showing optimal kinetic parameters in ovine AI doses [52]. Concerning the effect of time, our results showed a significant decrease in TM % and VSL at T2.5, regardless of condition. Despite this, TM % and VSL obtained at T2.5 were suitable for use in AI [52]. According to Gómez-Martín et al., [15], where Ma had no adverse effect on sperm quality in buck DS after 2.5 h post-contamination, our results also do not indicate any detrimental effect on sperm quality in C1 (DS + Ma) either. About SFD results, only the effect of time revealed significant results, showing an increase of SFD % (*p* < 0.01) between T0 and T15 of culture at 37 °C, regardless of the experimental condition. These results are similar to those obtained by Vicente-Fiel et al., [58], where an increase of SFD % was observed in ram semen after 6 h of incubation at 37 °C. Prolonged incubation, along with other well-known direct factors such as apoptosis, oxidative stress, or the presence of infectious agents, have been described as causes of sperm DNA fragmentation in rams and bulls [56, 89, 90]. Overall, these results confirm that the addition of P65 and L3 in ram DS would not motivate their discard due to poor sperm quality.

Metabarcoding analysis revealed a higher bacteria diversity in the richness index for C1 which increased significantly over time. This fact could be related to the pathogen’s viability, where a complete inhibition of Ma was observed at T15. This intrinsic bactericidal effect of DS would enable a richness increase in the natural and diverse microbiota present in ovine DS (Fig. 3). Moreover, C1 showed a higher DS volume compared with the other conditions (Table 2). Therefore, these differences in the composition of the conditions could influence the microbial diversity results. On the other hand, the conditions with lower richness were those containing P65 (C4 and C5). This lower richness is also reflected in the evenness results. The condition C4 showed the lowest Pielou’s index among all conditions at both time points. The reason of that could be explained highlighting the bacterial taxonomy of those conditions, where in the case of C4, a dominance of the species *E. hirae* is observed over time (RA in T0 = 98.64%; RA in T15 = 99.28%). This finding confirms the affinity of P65 for semen. Moreover, our metabarcoding results demonstrate for the first time that *E. hirae* species is a LAB, which naturally resides in the ovine seminal microbiota (C11) (Fig. 4). This also suggests its presence over other bacterial communities, specifically of the genus *Enterococcus* (C4 and C5), which may contribute to greater displacement of other bacteria communities, potentially displacing pathogenic species like Ma. Even in C1 (DS + Ma) at T15, after a total reduction in Ma viability, *E. hirae* showed a natural abundance increase. These metabarcoding data suggest that this bacterial species has an intrinsic relevant role in the control of seminal infections by Ma in sheep. In contrast, the conditions with L3, showed a more uniform bacterial diversity between the three commercial LAB strains, the general microbiota present in the DS and the pathogen.

The metabarcoding analysis revealed that the three species of *Lactobacillus* spp. present in the probiotic were inoculated in C2 and C3, which is consistent with previous studies [31]. No statistically significant differences for the RA among the three *Lactobacillus* species were observed. Regarding bacteria dynamics in the L3 inoculum, *L. crispatus* increased its abundance in the absence of Ma, contrasting with the findings of prior research [31]. In contrast, *L. acidophilus* decreased in abundance regardless of the pathogen’s presence. Conversely, *L. brevis* was less abundant overall but showed increased RA in the presence of Ma, confirming its ability to replicate in the presence of Ma [31]. These bacterial species have not been described in the prepuce or semen of small ruminants. Other identified species within this genus were *L. iners*, isolated from vaginal and seminal human samples [91, 92] and *L. rhamnosus* and *L. plantarum*, found in sheep dairy products with potential probiotic applications [93], and improving the creating of multi-strains probiotics in order to enhance their effectivity [94].

Disregarding the LAB, *A. seminis* was present in all conditions at both time points with high RA. This bacterial species has been frequently reported in preputial [21, 38] and seminal samples from rams [95]. Although *A. seminis* has been associated with abnormal semen quality and infertility in ovine [96] and present in herds with low AI success and abortions [21, 73], ram semen quality was not affected at the onset of the present study. Contrary to this, a significantly higher abundance of *A. seminis* in pregnant ewes compared to non-pregnant through natural mating was observed [38]. This bacterial species was the most abundant in C11, being detected for the first time in ram semen by metabarcoding. However, *A. seminis* showed a significantly greater decrease in abundance over time in all conditions (C3-C5) (*p* < 0.05), suggesting the use of LAB as a potential strategy for controlling antibiotic-resistant species like *A. seminis* [97]. To our knowledge, the most abundant genera previously reported in ovine commercial seminal doses with antibiotics were *Corynebacterium*, *Ureaplasma*, and *Pseudomonas* [21]. In contrast, our results identified *Actinobacillus*, *Lactobacillus*, and *Staphylococcus* as the most abundant ones. The abundance of *Lactobacillus* was similar to that reported for this genus in bucks ejaculate, where was higher during the non-breeding season [40]​. Thus, it appears that the addition of some antibiotics in the sperm extender during the preparation of semen doses could reduce the abundance of bacterial species sensitive to these antibiotics, such as LAB. Our findings provide the first data on the natural microbiota of ram DS without antibiotics. Notably, variability exists in the taxonomic profiles between samples, as factors such as season, environmental conditions, mating period, stress, and nutritional status can alter the microbial composition of semen in small ruminants [98]. Replacing antibiotics with LAB in semen extenders could help compensate for the loss of commensal bacteria in ejaculates caused by semen dilution. Moreover, given the risk of antimicrobial resistance associated with the preventive administration of antibiotics to females via semen doses at low concentrations, the inclusion of LAB in semen extenders may represent a promising strategy in the context of a potential future restriction on antibiotic use in small ruminants. This approach also addresses the serious problem of antimicrobial resistance associated with contagious agalactia [5], driven by the reduced antibiotic susceptibility of Ma to macrolides, lincosamides, tetracyclines, amphenicol, aminoglycosides and pleuromutilins [28]. Finally, the presence of *Staphylococcus*,* Streptococcus* and *Pseudomonas* (Fig. 3), previously described in preputial samples of rams [21, 38] were also present in our DS samples. Moreover, the presence of *Pseudomonas* and *Gardnerella* (Figs. 3 and 4) has also been correlated with normal sperm quality in humans [41]. Our results underline that the presence of LAB in the ejaculate of small ruminants could play an important role in bacterial reproductive ecology, justifying its addition to AI semen doses instead of antibiotics. Regarding the study’s limitations, the semen samples were intentionally pooled to minimize individual animal variability and obtain a representative composite sample for evaluating the treatment’s effects on sperm characteristics. Therefore, it’s important to note that this design doesn’t allow for the assessment of variation at the animal level, and a greater number of replicates would strengthen the generalizability of the results.

## Conclusions

Both lactic acid bacteria (LAB) inocula, one made of a commercial probiotic (L3) and the other of a small ruminant prepuce endogenous strain *Enterococcus hirae* P65, exhibited favourable growth in ram diluted semen and did not compromise the sperm quality required to produce seminal doses. Moreover, the addition of P65 to diluted semen, showed a great acidification capacity and ability to completely eliminate the *Mycoplasma agalactiae* presence. Although L3 showed antibacterial activity, its effect was significantly lower than strain P65. Moreover, in presence of P65, *Escherichia-Shigella* relative abundance did not increased over time. Their use would increase the quality of seminal doses by neutralising the potential presence of this pathogen and lowering the risk of seminal contamination by enterobacteria, therefore representing an alternative to antibiotics use in seminal doses. In addition, the acidification and antibacterial activity exhibited by diluted semen on its own could be due to the natural presence abundance of LAB in the ejaculate of small ruminants, that could be affected by dilution with sperm extenders and the addition of antibiotics. Considering the possible beneficial effects of LAB and the absence of negative impact on sperm quality, our study also proposes, for the first time, the use of seminal fluid as a vehicle for the intravaginal inoculation of LAB in small ruminants. Despite this, there is a necessity of in vivo studies to evaluate the effect in sheep flocks of these probiotics on fertility and, naturally circulating sexual pathogens such mycoplasmas, for an overall improvement of animal welfare.

## Supplementary Information

Supplementary Material 1.

Supplementary Material 2.

## Data Availability

All data generated or analysed during this study are included in this published article and its supplementary information files. Additionally, the datasets generated and analysed during the current study are available in the European Nucleotide Archive (ENA) (European Molecular Biology Laboratory, European Bioinformatics Institute (EMBL-EBI): https://www.ebi.ac.uk/ena/browser/view/PRJEB95893, with the study BioProject ID PRJEB95893. The full genome sequence of strain P65 can be found in the GenBank repository (ERR15902943 submission number).
