# Supplementary material for: Addition of lactic acid bacteria to diluted ram semen as vehicle for vaginal inoculation: interaction with seminal microbiota, sperm quality and antibacterial in vitro effect against Mycoplasma agalactiae
Source: BMC Vet Res. 2026 May 6;22:369. doi: 10.1186/s12917-026-05536-2 (PMC13317260; doi:10.1186/s12917-026-05536-2)
Supplement: Supplementary file 1 — Supplementary Material 1. [file 12917_2026_5536_MOESM1_ESM.docx]

**Supplementary Table S1.** Median (interquartile interval) of the alpha diversity indices of experimental conditions over time.

| Comparison | Alpha diversity metric | | |
| --- | --- | --- | --- |
| C1 | Richness  (Observed ASV´s) | *P*-value | 0.00585** |
|  |  | T0 | 8.67 |
|  |  | T15 | 15 |
|  | Evenness  (Pielou index) | *P*-value | 0.91473 |
|  |  | T0 | 0.32 |
|  |  | T15 | 0.35 |
| C2 | Richness  (Observed ASV´s) | *P*-value | 0.80978 |
|  |  | T0 | 6.67 |
|  |  | T15 | 7 |
|  | Evenness  (Pielou index) | *P*-value | 0.04168* |
|  |  | T0 | 0.68 |
|  |  | T15 | 0.37 |
| C3 | Richness  (Observed ASV´s) | *P*-value | 0.41284 |
|  |  | T0 | 8.33 |
|  |  | T15 | 9.67 |
|  | Evenness  (Pielou index) | *P*-value | 0.5401 |
|  |  | T0 | 0.67 |
|  |  | T15 | 0.77 |
| C4 | Richness  (Observed ASV´s) | *P*-value | 0.71201 |
|  |  | T0 | 3 |
|  |  | T15 | 2.67 |
|  | Evenness  (Pielou index) | *P*-value | 0.5915 |
|  |  | T0 | 0.06 |
|  |  | T15 | 0.04 |
| C5 | Richness  (Observed ASV´s) | *P*-value | 0.55223 |
|  |  | T0 | 4.67 |
|  |  | T15 | 4 |
|  | Evenness  (Pielou index) | *P*-value | 0.09892 |
|  |  | T0 | 0.45 |
|  |  | T15 | 0.29 |

**P-value* < 0.05; ** *P-value* < 0.01. C1: condition 1 with diluted semen and *Mycoplasma agalactiae* PG2; C2: condition 2 with diluted semen and L3; C3: condition 3 with diluted semen, *Mycoplasma agalactiae* PG2 and L3; C4: condition 4 with diluted semen, *Mycoplasma agalactiae* PG2 and P65; C5: condition 5 with diluted semen, *Mycoplasma agalactiae* PG2 and P65; T0: 15 minutes after incubation; T15: 15 hours after incubation.

**Supplementary Table S2.** Median (interquartile interval) of the alpha diversity indices of experimental time points depending on experimental conditions.

| Comparison | Alpha diversity metric | | | *P-value* |
| --- | --- | --- | --- | --- |
| C1_C2 | Richness  (Observed ASV´s) | T0 (C1_C2) | 8.67_6.67 | 0.92148 |
|  |  | T15 (C1_C2) | 15_7 | 0.17488 |
|  | Evenness  (Pielou index) | T0 (C1_C2) | 0.32_0.68 | 0.46827 |
|  |  | T15 (C1_C2) | 0.35_0.37 | 0.99999 |
| C1_C3 | Richness  (Observed ASV´s) | T0 (C1_C3) | 8.67_8.33 | 0.99985 |
|  |  | T15 (C1_C3) | 15_9.67 | 0.58555 |
|  | Evenness  (Pielou index) | T0 (C1_C3) | 0.32_0.67 | 0.11174 |
|  |  | T15 (C1_C3) | 0.35_0.78 | 0.02605* |
| C1_C4 | Richness  (Observed ASV´s) | T0 (C1_C4) | 8.67_3 | 0.07959 |
|  |  | T15 (C1_C4) | 15_2.67 | 0.00454** |
|  | Evenness  (Pielou index) | T0 (C1_C4) | 0.32_0.06 | 0.00245** |
|  |  | T15 (C1_C4) | 0.35_0.01 | 0.00006** |
| C1_C5 | Richness  (Observed ASV´s) | T0 (C1_C5) | 8.67_4.67 | 0.40078 |
|  |  | T15 (C1_C5) | 15_4 | 0.01698* |
|  | Evenness  (Pielou index) | T0 (C1_C5) | 0.32_0.45 | 0.95093 |
|  |  | T15 (C1_C5) | 0.35_0.29 | 0.98404 |
| C2_C3 | Richness  (Observed ASV´s) | T0 (C2_C3) | 6.67_8.33 | 0.96122 |
|  |  | T15 (C2_C3) | 7_9.67 | 0.85687 |
|  | Evenness  (Pielou index) | T0 (C2_C3) | 0.68_0.67 | 0.93154 |
|  |  | T15 (C2_C3) | 0.37_0.78 | 0.03314* |
| C2_C4 | Richness  (Observed ASV´s) | T0 (C2_C4) | 6.67_3 | 0.24647 |
|  |  | T15 (C2_C4) | 7_2.67 | 0.13622 |
|  | Evenness  (Pielou index) | T0 (C2_C4) | 0.68_0.06 | 0.00003** |
|  |  | T15 (C2_C4) | 0.37_0.01 | 0.00005** |
| C2_C5 | Richness  (Observed ASV´s) | T0 (C2_C5) | 6.67_4.67 | 0.82824 |
|  |  | T15 (C2_C5) | 7_4 | 0.51875 |
|  | Evenness  (Pielou index) | T0 (C2_C5) | 0.68_0.45 | 0.8863 |
|  |  | T15 (C2_C5) | 0.37_0.29 | 0.9728 |
| C3_C4 | Richness  (Observed ASV´s) | T0 (C3_C4) | 8.33_3 | 0.09914 |
|  |  | T15 (C3_C4) | 9.67_2.67 | 0.03375 |
|  | Evenness  (Pielou index) | T0 (C3_C4) | 0.67_0.06 | 0* |
|  |  | T15 (C3_C4) | 0.78_0.01 | 0* |
| C3_C5 | Richness  (Observed ASV´s) | T0 (C3_C5) | 8.33_4.67 | 0.47669 |
|  |  | T15 (C3_C5) | 9.67_4 | 0.14935 |
|  | Evenness  (Pielou index) | T0 (C3_C5) | 0.67_0.45 | 0.42767 |
|  |  | T15 (C3_C5) | 0.78_0.29 | 0.00534** |
| C4_C5 | Richness  (Observed ASV´s) | T0 (C4_C5) | 3_4.67 | 0.74965 |
|  |  | T15 (C4_C5) | 2.67_4 | 0.81878 |
|  | Evenness  (Pielou index) | T0 (C4_C5) | 0.06_0.45 | 0.00037** |
|  |  | T15 (C4_C5) | 0.01_0.29 | 0.00036** |

**P-value* < 0.05; ** *P-value* < 0.01. ASVs, amplicon sequence variant. C1: condition 1 with diluted semen and *Mycoplasma agalactiae* PG2; C2: condition 2 with diluted semen and L3; C3: condition 3 with diluted semen, *Mycoplasma agalactiae* PG2 and L3; C4: condition 4 with diluted semen, *Mycoplasma agalactiae* PG2 and P65; C5: condition 5 with diluted semen, *Mycoplasma agalactiae* PG2 and P65; T0: 15 minutes after incubation; T15: 15 hours after incubation.

**Supplementary Table S3.** FDR-adjusted *P* values of pairwise comparisons using PERMANOVA analysis for beta diversity indexes matrices.

| Variables | Qualitative indexes | | Quantitative indexes | |
| --- | --- | --- | --- | --- |
|  | Jaccard | Unweighted UniFrac | Bray Curtis | Weighted UniFrac |
| Time (T0_T15) | 0.203 | 0.658 | 0.249 | 0.036* |
| Condition_Time | 0.544 | 0.605 | 0.322 | 0.31 |

**P-value* < 0.05. T0: 15 minutes after incubation; T15: 15 hours after incubation.
