# Supplementary figures and images for: Addition of lactic acid bacteria to diluted ram semen as vehicle for vaginal inoculation: interaction with seminal microbiota, sperm quality and antibacterial in vitro effect against Mycoplasma agalactiae
Source: BMC Vet Res. 2026 May 6;22:369. doi: 10.1186/s12917-026-05536-2 (PMC13317260; doi:10.1186/s12917-026-05536-2)

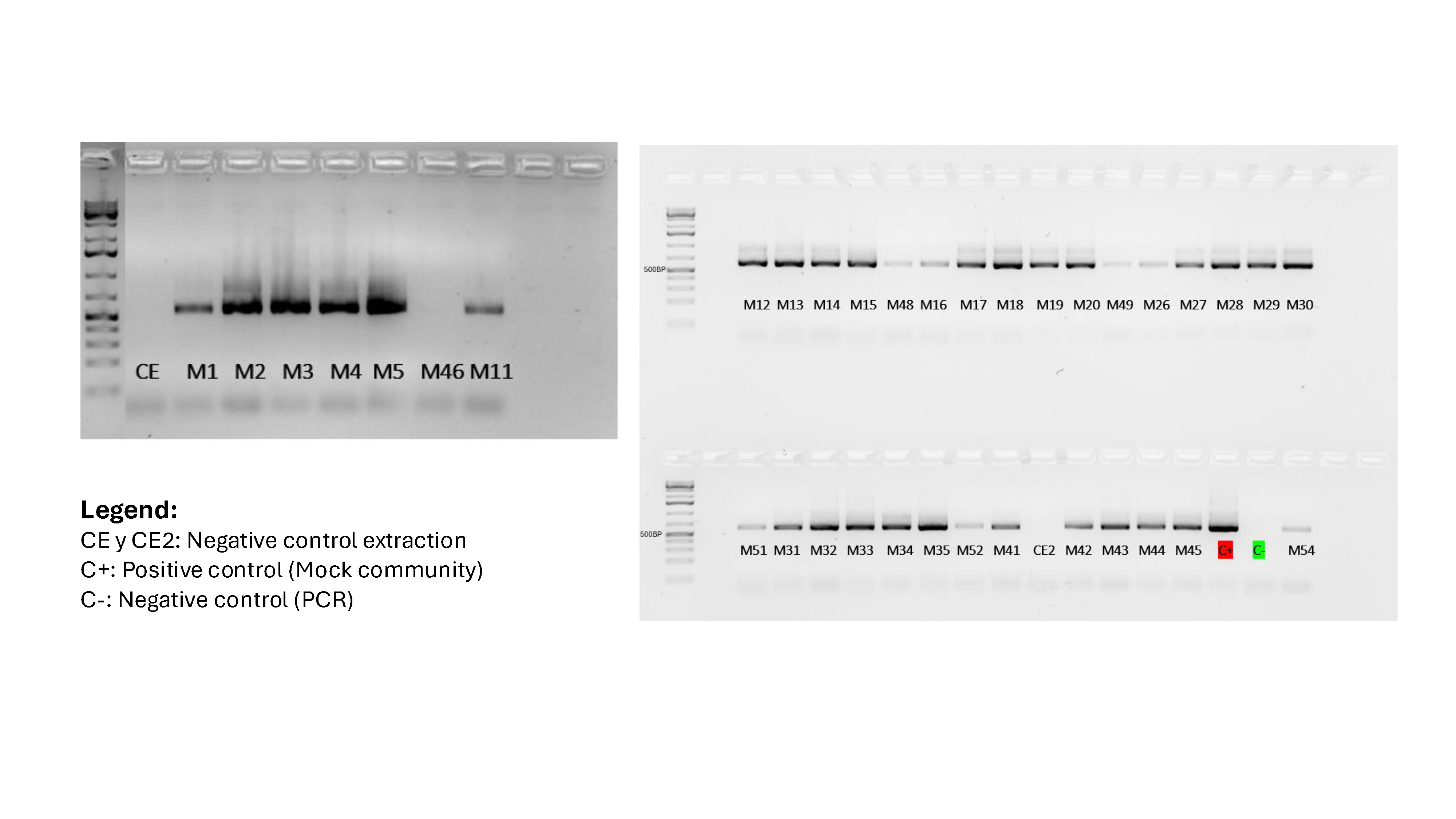

Supplement: Supplementary file 2 — Supplementary Material 2. [file 12917_2026_5536_MOESM2_ESM.png]
